# Impact of Preoperative Three-Dimensional Computed Tomography Cholangiography on Postoperative Resection Margin Status in Patients Operated due to Hilar Cholangiocarcinoma

**DOI:** 10.1155/2017/1947023

**Published:** 2017-08-16

**Authors:** A. Andert, P. Bruners, C. Heidenhain, F. Ulmer, C. D. Klink, P. H. Alizai, C. Kuhl, U. P. Neumann, M. Binnebösel

**Affiliations:** ^1^Department of General, Visceral and Transplantation Surgery, RWTH Aachen University Hospital, Aachen, Germany; ^2^Department of Diagnostic and Interventional Radiology, RWTH Aachen University Hospital, Aachen, Germany; ^3^Department of General and Visceral Surgery, Sana Hospital Düsseldorf-Gerresheim, Düsseldorf, Germany; ^4^Department of Surgery, Maastricht University Medical Centre, Maastricht, Netherlands

## Abstract

**Introduction:**

The purpose of this study was to analyse the value of 3-dimensional computed tomography cholangiography (3D-ERC) compared to conventional retrograde cholangiography in the preoperative diagnosis of hilar cholangiocarcinoma (HC) with special regard to the resection margin status (R0/R1).

**Patients and Methods:**

All hepatic resections performed between January 2011 and November 2013 in patients with HC at the Department of General, Visceral and Transplant Surgery of the RWTH Aachen University Hospital were analysed. All patients underwent an ERC and contrast-enhanced multiphase CT scan or a 3D-ERC.

**Results:**

The patient collective was divided into two groups (group ERC: *n* = 17 and group 3D-ERC: *n* = 16). There were no statistically significant differences between the two groups with regard to patient characteristics or intraoperative data. Curative liver resection with R0 status was reached in 88% of patients in group ERC and 87% of patients in group 3D-ERC (*p* = 1.00). We could not observe any differences with regard to postoperative complications, hospital stay, and mortality rate between both groups.

**Conclusion:**

Based on our findings, preoperative imaging with 3D-ERC has no benefit for operative planning and R0 resection status. It cannot replace the exploration by an experienced surgeon in a centre for hepatobiliary surgery.

## 1. Introduction

For patients with hilar cholangiocarcinoma (HC), liver surgery is the only chance for a cure thus far. The surgical treatment of HCs has improved over the last decades. Initially, isolated resections of the extrahepatic bile duct were performed. Eventually, extended liver resections combined with resection of the portal vein and sometimes even the hepatic artery became the gold standard. Among clinical trials, even liver transplantation could be a treatment option in some cases [1–3]. However, the rates of curative resections are still unsatisfactory and 5-year survival rates are below 40% [4–7].

One fundamental problem is to obtain an accurate diagnosis of longitudinal and vertical tumour spreading on the basis of the preoperative imaging. HCs are located in the hepatic hilum with a close relationship to the portal vein and hepatic artery. Visualization and identification of HCs by separation between tumour and the surrounding tissue is complex because HCs do not present as a solid tumour mass. Despite exhaust radiographic and interventional diagnostic in most cases, an exploration has to be performed for a precise estimation of the tumour expansion [8]. On the one hand, precise preoperative diagnostic is fundamental for an optimal planning of the operation, and on the other hand, a R0 resection is the most important fact predicting a long-term patient survival [9].

Different diagnostic tools are available for preoperative imaging, all with their own strengths and weaknesses: endoscopic retrograde cholangiography (ERC), magnetic resonance imaging (MRI) with cholangiography (MRC), computed tomography (CT), or percutaneous transhepatic cholangiography (PTC) [10].

MRI with MRCP can visualize bile ducts, vessels, and the liver parenchyma. It is a noninvasive procedure, and patients are not exposed to radiation. However, pictures of vascular invasion by MRI are still inferior to those of multidetector row (MDR) CT, and evaluation of lymph node metastasis is less feasible on MRI because of the low spatial resolution of the technique [11]. Furthermore, biliary stents may cause susceptibility artifacts.

CT has good sensitivity and specificity for the evaluation of portal vein and hepatic artery involvement and is used as staging imaging for metastasis and lymph node status [12]. ERC shows adequate prediction for horizontal tumour spread and has the advantage of obtaining tissue for histological examination and acting interventionally, for example, inserting a stent in the case of cholestasis. The latter may be very useful in the case of preoperative functional preconditioning of the remaining liver segments. The combination of 3-dimensional CT scan and ERC (3D-ERC) seems to be a reliable method for an accurate preoperative diagnosis of horizontal and vertical tumour extent. Thus, the operative strategy and resection line could be evaluated preoperatively more exactly and the prediction rate of R0 resection may be higher.

Therefore, the purpose of this study was to analyse the value of 3-dimensional computed tomography cholangiography (3D-ERC) compared to conventional retrograde cholangiography in the preoperative diagnostic for hilar cholangiocarcinoma with special regard to the resection margin status (R0/R1).

## 2. Patients and Methods

All hepatic resections that were performed between January 2011 and November 2013 in patients with HC at the Department of General, Visceral and Transplant Surgery of the RWTH Aachen University Hospital were prospectively registered in a database, and data of these patients were retrospectively analysed. Patients with HC Bismuth type I were excluded because they did not receive 3D-ERC for operative planning. Within the preoperative diagnostic, all patients underwent an ERC and contrast-enhanced multiphase CT scan. Additionally, MRI with MRC was performed in 50% of patients.

3D-ERC was performed in all patients who were introduced to our department without or with an inadequate conventional ERC or without an effective bile duct drainage as explained below. Due to the risk of post-ERC pancreatitis or cholangitis, ERC and/or 3D-ERC was not performed in patients that were introduced to our department with a proper ERC imaging and/or a sufficient drainage of cholestatic bile ducts.

The Ethics Committee of the RWTH Aachen University Hospital approved this retrospective study (EK 283/16). All patients signed a risk consent form preoperatively, including acceptance to receive preoperative planning with ERC or 3D-ERC.

### 2.1. Three-Dimensional Computed Tomography Cholangiography

During standard ERC in prone position under X-ray fluoroscopy, a nasobiliary probe was inserted in the common bile duct. Under sedation and analgesia, patients were transferred from a standard X-ray table to the CT table. After placing patients in the supine position, a 1 : 10 dilution of iopromid (Ultravist 370, Bayer Vital, Leverkusen, Germany) was manually injected via the catheter located in the common bile duct. The volume of contrast solution needed to obtain opacification of the whole biliary tree was estimated on the basis of the preceding ERC. After contrast administration, a CT-scan was performed with the following parameters: collimation 64 × 0.6 mm, 120 kV tube voltage, and 330 ms rotation time. Axial and coronal image data sets were reconstructed with a slice thickness of 1 and 5 mm and an increment of 0.7 mm and 3 mm, respectively. In addition, volume rendering reconstructions (VRT) and maximum intensity projections (MIP) were obtained and compared to the standard ERC projections.

### 2.2. Preoperative Treatment and Surgical Approach

Preoperative cholestasis was treated by ERC with stent or percutaneous transhepatic cholangio-drainage. It was obligatory in all cases with cholestasis within the remaining liver lobe to drain all segmental bile ducts of these liver segments. A serum bilirubin level of less than 5 mg/dl was used as a trigger for the timing of the operation.

**Figure 1:**
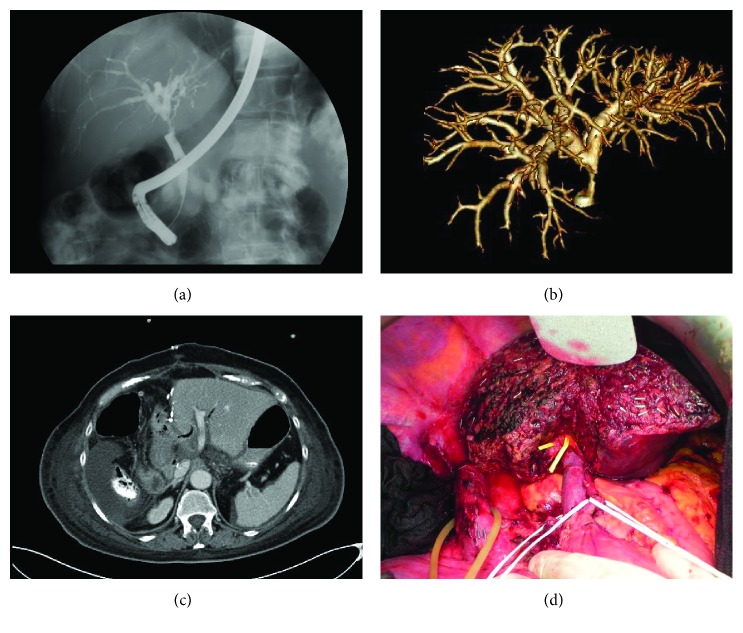
Preoperative imaging and intra- and postoperative status after hepatectomy. (a) Conventional ERC, patient with Klatskin tumor bismuth type II. (b) 3D-ERC, patient with Klatskin tumor bismuth type II. (c) Axial CT scan 4 days after extended right hepatectomy. (d) Intraoperative status after right trisectionectomy.

Surgical techniques as well as intraoperative and postoperative care were standardised. Abdominal access was obtained via a midline incision with right lateral subcostal incisions. All resections were performed as hilar en bloc resections with dissection of the regional lymph nodes in the ligamentum hepatis and the upper edge of the pancreas up to the celiac trunk. Hepatic dissection was performed with a Cavitron Ultrasonic Surgical Aspirator (CUSA, Integra, Germany). Vessels and bile ducts located in the parenchyma were closed with clips. All operations were performed following preoperative planning. Postoperative patients were monitored in the ICU for at least one night. The following liver resections were performed: extended right hepatectomy (segments 1, 5, 6, 7, and 8), extended left hepatectomy (segments 1, 2, 3, and 4), right trisectionectomy (segments 1, 4, 5, 6, 7, and 8), and left trisectionectomy (segments 1, 2, 3, 4, 5, and 8).  Figure 1 shows the preoperative imaging with ERC and 3D-ERC as well as the intra- and postoperative status after hepatectomy.

In cases where the future remnant liver was <30%, percutaneous embolisation of the contralateral branches of the portal vein was performed 14–28 days prior to surgery. Hepatic volume was measured before and after portal vein embolisation using contrast-enhanced computed tomographic volumetry.

The endpoint of the study was the histopathological resection margin status (R0 versus R1) in accordance with the preoperative diagnostic with ERC or 3D-ERC. Data were collected with respect to age, gender, tumour localization according to the Bismuth classification, type of liver resection, and operative time. Laboratory values as bilirubin (mg/dl), gamma glutamyl tranferase (*γ*GT) (U/l), alkaline phosphatase (AP) (U/l), lipase (U/l), and amylase (U/l) were measured before ERC or 3D-ERC and 24 h after the intervention as well as before operation and on postoperative days 1, 2, 3, 4, and 7.

Pathology results including the TNM classification and *R* status were analysed. The outcome of hepatic resection in both groups was evaluated by the length of postoperative hospital and intensive care unit (ICU) stay, postoperative complications, and in-hospital mortality. Preoperative cholangitis was present, when patients suffered from fever, increased WBC count/CRP, and GGT/AP. Post-ERC or 3D-ERC pancreatitis was defined as serum amylase or lipase levels that were three times higher than normal serum concentrations. Bile leakage was defined as a bilious collection with bilirubin concentration in the drain fluid that was three times higher than the serum concentration. Abscesses were defined as nonbilious fluid collections requiring CT-guided drainage or surgery. Postresectional liver failures (PRLF) were classified according to the definition and grading by the International Study Group of Liver Surgery (ISGLS) [13].

### 2.3. Statistic

Statistical analyses were performed using SPSS statistical software (IBM®, SPSS® Statistics 20, Chicago, IL, USA). The chi-square test or Fisher's exact test was applied for qualitative variables and the Mann–Whitney test for continuous variables. A two-sided *p* value of <0.05 was considered to be significant. For continuous variables, the results are provided as the median and range (minimum and maximum).

## 3. Results

Between January 2011 and November 2013, 33 hepatic resections were performed in patients with HC Bismuth type II–IV. The patient collective was divided into two groups. Group ERC included 17 patients and group 3D-ERC included 16 patients.

### 3.1. Patient Characteristics

There were no statistically significant differences between both groups with regard to patient characteristics. The median age was 69 in group ERC and 70 in group 3D-ERC. In group ERC, 53% of patients were male and 47% female. In group 3D-ERC, 81% of patients were male and 19% female. The main indication for surgery was a Klatskin tumour Bismuth type III. Portal vein embolisation prior to surgery was performed in 47% of patients in group ERC and 63% in group 3D-ERC. Mild post-ERC pancreatitis was observed in only one case in group ERC and in none of the patients following 3D-ERC. At the time of operation, all patients had bilirubin levels < 5 mg/dl. In group 3D-ERC, 56% of patients suffered from cholangitis preoperatively (tumour associated) and in group ERC 29%. Detailed clinical characteristics are presented in Table 1.

### 3.2. Intraoperative Data

No statistically significant difference was found between the two groups with respect to the intraoperative data. The most frequently performed operation was a right trisectionectomy in both groups (41% in group ERC and 43% in group 3D-ERC). Portal vein resection was performed in 53% of patients in group ERC and 50% of patients in group 3D-ERC. Portal vein resection combined with resection of the hepatic artery was necessary in 12% of patients in group ERC and 19% of patients in group 3D-ERC. The median operation time was 341 minutes in group ERC and 329 minutes in group 3D-ERC.  Table 2 shows a complete survey of the intraoperative data.

### 3.3. Hospital Stay and Postoperative Complications

The median postoperative hospital stay was 20 days in group ERC and 27 days in group 3D-ERC. The mean ICU stay was 2 days in both groups (Table 3).

The number of patients with wound infections, pneumonia, renal failure with the need for temporary haemodialysis, and sepsis was comparable and without statistically significant differences in both groups. Furthermore, we could not observe significant differences of liver-related complications comparing both groups. Postoperative complications are depicted in Table 3.

### 3.4. Resection Status

Curative liver resection with R0 status was reached in 88% of patients in group ERC and 87% of patients in group 3D-ERC group (*p* = 1.00). Two patients in group ERC had an R1 resection, one because of infiltration of the portal vein and one because of infiltration of the bile duct margin in the final pathology report. In group 3D-ERC, 2 patients had an R1 resection due to infiltration of the bile duct in the final pathology report and the liver resection surface.

## 4. Discussion

The aim of our study was to examine the value of 3-dimensional computed tomography cholangiography (3D-ERC) compared to conventional retrograde cholangiography in the preoperative diagnostic for hilar cholangiocarcinoma with special regard to the resection margin status (R0/R1).

The technology of three-dimensional reconstruction of CT scans was introduced several years ago. Meanwhile, different procedures for CT cholangiography have arisen [14–17]. However, 3D-ERC does not belong to the standard diagnostic in HC, and to our knowledge, only few other studies have investigated the value of 3D-ERC for operative planning in patients with HC [18–22].

The focus of our investigation was the resection margin status. Independently of the preoperative diagnostic, the R0 resection rate in patients with HC generally varies from 14% to 80% [23, 24]. In our population, we had an overall R0 resection rate of 88% and we could not show any statistically significant difference between both groups.

In our study collective, two cases of R1 resection were due to a tumour infiltration at the bile duct margin. In both cases, the bile duct margin was negative by intraoperatively performed frozen section examination, and further resection had been possible. In the other cases, infiltrations of the portal vein or liver resection surface were the reasons for R1 resection. A further resection was technically not possible in both patients. The problem of false-negative results quantified by intraoperative performed frozen section examinations is known and has been explained by Mantel and Lim before. Mantel et al. investigated the accuracy of intraoperative frozen section analysis of the proximal bile ducts in patients with HC. They showed that intraoperative frozen section analysis is of limited value because of low sensitivity (68%) and a high rate of false-negative results (16%) [25]. Lim and Park explained the impaired sensitivity of intraoperative frozen section analysis in HC due to the specific growth pattern of the tumor with a longitudinal, infiltrative extension along the mucosa and submucosa [26]. Therefore, the challenge of accurate diagnostic of tumor extensiveness consists up to a final histopathological examination.

Bartsch et al. published their data of 96 patients who underwent liver resection for HC. The preoperative evaluation was performed by either a conventional CT scan or an MRI. They reached an R0 resection in 78% of patients. They advocated that the limit of surgical resection for bile duct cancer is the advanced stage of the tumour (T stadium). Whereas in a T3 stadium an R0 resection was possible in most cases, they were unable to perform an R0 resection in a T4 stadium. From their perspective, the T stadium cannot be estimated through expanded preoperative diagnostics but only through surgical exploration [8].

On the other hand, some studies revealed the accuracy of 3D imaging in matters of the resection margin status [19–22, 27]. Sasaki et al. conducted a prospective study on the utility of 3D analysis with multidetector row (MDR) CT cholangioportography in operative planning for HC. They could predict the ductal margin status correctly in 17 of 18 cases [20]. Because they did not have a control group, the advantage of 3D MDR CT could not be noted clearly. Endo et al. analysed preoperative and intraoperative findings of 15 patients who underwent preoperative 3D MDR CT cholangiography. The accuracy for longitudinal tumour extension was 85% for cholangiography alone and 87% for 3D cholangiography. In 14 of 15 patients, a curative resection was achieved [21].

Kim et al. performed a prospective study with 11 patients with HC who underwent contrast material-enhanced MDR CT cholangiography. In 10 of the 11 patients, the correct determination of tumour extent was possible [22]. They did not mention the *R* status, and the advantage of 3D MDR CT was not clear because of the missing control group. Furukawa et al. reported their results of 3D direct CT cholangiography in 5 patients with HC. They described a diagnostic accuracy of 100%. The resected margin was free from tumour in all cases [27]. Nagakawa et al. fused conventional 3-dimensional computed tomography with multiplanar reconstruction images and peroral cholangioscopy findings for preoperative assessment of HC. They reached an R0 resection in 10 out of 12 patients and therefore described a diagnostic accuracy for horizontal tumour spread of 83.3% [19]. Of note, with normal MDR CT, the preoperative diagnosis was correct in 9 of 12 patients.

Though 3D illustration of the bile duct anatomy may be of importance for operative planning. However, in comparison to conventional ERC and/or MRI/MRC, 3D-ERC showed no benefit to the R0 resection rate.

Furthermore, it is associated with a remarkable organizational effort and additional costs. Three-dimensional imaging cannot replace the exploration by an experienced surgeon in a centre for hepatobiliary surgery.

Some limitations exist concerning the evaluation of our data. First, this analysis was performed retrospectively. Furthermore, the number of patients in both groups was relatively small. Multivariate analyses in larger patient collectives need to be performed to categorise the impact of 3D-ERC in the context of preoperative imaging for hilar cholangiocarcinoma.

## 5. Conclusions

Based on our findings, preoperative imaging with 3D-ERC had no statistically significant benefit with regard to R0 resection status. Three-dimensional imaging cannot replace the exploration by an experienced surgeon in a centre for hepatobiliary surgery.

## Figures and Tables

**Table 1:** Patient characteristics.

Patient characteristics	ERC, *n* = 17	3D-ERC, *n* = 16	*p* value
Gender			0.080
Male	9 (53%)	13 (81%)	
Female	8 (47%)	3 (19%)	
Age	69 (58–79)	70 (50–81)	0.804
Bismuth classification			0.634
Type II	2 (12%)	1 (6%)	
Type IIIa	6 (35%)	7 (44%)	
Type IIIb	6 (35%)	3 (19%)	
Type IV	3 (18%)	5 (31%)	
*Preoperative MRI with MRC*	3 (18%)	5 (31%)	0.283
Post-ERC pancreatitis	1 (11%)	0	0.360
Preoperative cholangitis	5 (29%)	9 (56%)	0.166

**Table 2:** Intraoperative data.

Intraoperative data	ERC, *n* = 17	3D-ERC, *n* = 16	*p* value
Type of liver resection			0.721
Right extended hepatectomy	4 (24%)	5 (31%)	
Left extended hepatectomy	5 (29%)	2 (13%)	
Right trisectionectomy	7 (41%)	7 (43%)	
Left trisectionectomy	1 (6%)	2 (13%)	
Extent of vascular resection			1
Portal vein	9 (53%)	8 (50%)	
Portal vein and hepatic artery	2 (12%)	3 (19%)	
Operation time (min)	341 (243–567)	329 (262–404)	0.683
Resection margin R1	2 (12%)	2 (13%)	1

**Table 3:** Postoperative complications and hospital stay.

Postoperative data	ERC, *n* = 17	3D-ERC, *n* = 16	*p* value
Liver specific complications			
Biliary leakage	2 (12%)	6 (38%)	0.118
Intraabdominal abscess	1 (6%)	5 (31%)	0.085
Intraabdominal haemorrhage	1 (6%)	2 (13%)	0.601
Liver failure grade B	1 (6%)	2 (13%)	0.601
Other complications			
Wound infection	2 (12%)	6 (38%)	0.118
Pneumonia	1 (6%)	0	1
Renal failure with hemodialysis	1 (6%)	3 (19%)	0.335
Sepsis	1 (6%)	3 (19%)	0.335
Postoperative hospital stay (days)	20 (11–82)	27 (8–72)	0.191
ICU stay (days)	2 (2–38)	2 (1–23)	0.988
